# Neutrophil Elastase Inhibitors: A potential prophylactic treatment option for SARS-CoV-2-induced respiratory complications?

**DOI:** 10.1186/s13054-020-03023-0

**Published:** 2020-06-08

**Authors:** Mahmoud M. A. Mohamed, Ismail Amr El-Shimy, Muhammad Abdul Hadi

**Affiliations:** 1 Department of Radiology, Charité - Universitätsmedizin Berlin, Charitéplatz 1, 10117 Berlin, Germany; 2 Integrative Research Institute (IRI) for Life Sciences, Humboldt University Berlin, Philippstrasse 13, 10115 Berlin, Germany; 3 Institute of Pathology, Charité - Universitätsmedizin Berlin, Charitéplatz 1, 10117 Berlin, Germany; 4 School of Pharmacy, College of Medical and Dental Sciences, University of Birmingham, Edgbaston, Birmingham, B15 2TT UK

As the death toll from the COVID-19 pandemic caused by SARS-CoV-2 continues to mount globally, scientists, healthcare agencies, and pharmaceutical companies are trying hard to find a “cure” and devise treatment strategies to reduce mortality. “Repurposing” existing drugs to fight COVID-19 remains an important strategy. Since respiratory failure remains one of the leading causes of death in COVID-19 patients, in this commentary, we have critically discussed the potential benefit of neutrophil elastase inhibitors (NEIs) in patients hospitalised with severe COVID-19.

Around one in three COVID-19 patients admitted to a hospital develop systemic inflammatory conditions such as cytokine release syndrome (CRS) [1] and acute respiratory distress syndrome (ARDS) [2]. Since lymphocytopenia is often reported in severe COVID-19 patients, it suggests that systemic inflammatory complications, associated with disease severity and mortality, are likely to be mediated by leukocytes other than T cells [3]. The drop in lymphocyte count is accompanied by an increase in neutrophil count and a decrease in monocytes, eosinophils, and basophils [4, 5], indicating that, together with lymphocytopenia, increased neutrophil count and neutrophil-to-lymphocyte ratio may be important predictors of disease severity in COVID-19 patients [5]. A recent case study supported this hypothesis [6]. The patient’s deterioration on day 12 of illness was preceded by an elevation in his neutrophil count on day 11, while lymphocytes and monocytes remained low [6]. Given that there is often a short window between the time of hospital admission and development of ARDS in severe cases [3, 6], a rapid prophylactic therapy is warranted to effectively prevent complications and death.

Neutrophils play a pivotal role in the development of ARDS through the production of toxic mediators including reactive oxygen species and proteases, especially elastase [7]. Furthermore, neutrophils can produce interleukin 6 (IL-6) in response to viral infections, in particular single-stranded RNA viruses such as SARS-CoV-2 via a Toll-like receptor 8 (TLR8)-mediated mechanism [8]. These cells are also important sources of soluble IL-6 receptors (IL-6R) in the lungs and may contribute to pathogenic IL-6R trans-signaling in chronic respiratory diseases [9]. The importance of this kind of signaling for the development of CRS has been demonstrated in chimeric antigen receptor T cell (CART)-treated lymphoma patients [10]. These studies suggest that increased neutrophil count can contribute to CRS and lung damage in patients with ARDS. Additionally, elastase secreted by these cells is one of the key proteolytic enzymes shown to activate the spike (S) protein of coronaviruses and shift the viral entry to a low pH-independent route [11].

We advocate the use of NEIs such as sivelestat to alleviate neutrophil-induced damage in high-risk COVID-19 patients. Initiation of sivelestat will serve two strategic purposes; first, it will mitigate the damaging effect of neutrophil elastase on the lung connective tissue, and second, it will limit the virus spreading capabilities by preventing S protein proteolytic activation (Fig. 1). Sivelestat is approved in Japan and the Republic of Korea for the treatment of acute lung injury and ARDS. Although existing clinical data is somewhat conflicting, the severity of lung injury remains an important predictor for treatment outcomes in such patients [12, 13]. Clinical trials that reported positive outcomes of sivelestat treatment in patients with ARDS and ALI had recruited patients mainly with lung injury score (LIS) < 2.5. On the other hand, trials reporting negative outcomes particularly the STRIVE study had recruited patients mainly with LIS > 2.5 [12, 13] emphasizing the critical importance of an early intervention with sivelestat. Notably, patients enrolled in the STRIVE study were more heterogeneous than the other trials and included more cases with non-pulmonary organ failures, conditions that are not relevant to COVID-19 patients [12, 13]. Furthermore, post hoc analysis of patient subgroups from the STRIVE study with mean LIS < 2.5 and those with systemic inflammatory response syndrome revealed a positive outcome of sivelestat on mortality rate and ventilator-free days [12, 13]. More importantly, the STRIVE study failed to identify any evidence of drug-related toxicity and did not offer any plausible explanation for the increase in long-term mortality in sivelestat-treated groups [12].

**Fig. 1:**
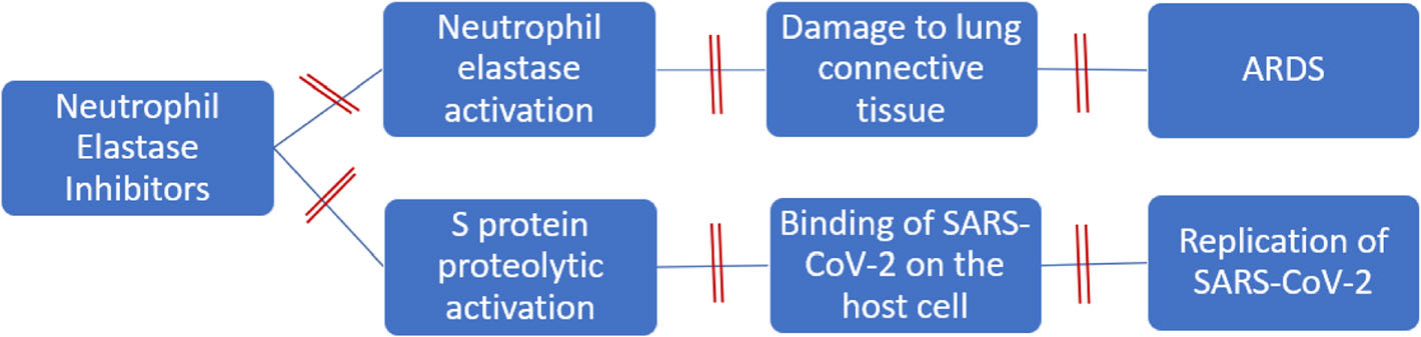
Mechanism of action of neutrophil elastase inhibitors in COVID-19.  indicates block. ARDS acute respiratory distress syndrome

Although current evidence to support the use of NEIs in ARDS induced by COVID-19 is lacking, we hypothesize that early administration of these drugs to patients with lymphocytopenia and LIS < 2.5 may be of significant value to prevent disease progression. Future clinical trials should be designed to evaluate the effectiveness of sivelestat in COVID-19 patients admitted to hospital with high risk of respiratory failure.

## Data Availability

Not applicable
